# Copper-Dependent Kinases and Their Role in Cancer Inception, Progression and Metastasis

**DOI:** 10.3390/biom12101520

**Published:** 2022-10-20

**Authors:** Alessandra Vitaliti, Anastasia De Luca, Luisa Rossi

**Affiliations:** 1 PhD Program in Cellular and Molecular Biology, Department of Biology, University of Rome “Tor Vergata”, Via della Ricerca Scientifica 1, 00133 Rome, Italy; 2 Department of Biology, University of Rome “Tor Vergata”, Via della Ricerca Scientifica 1, 00133 Rome, Italy

**Keywords:** copper, copper-dependent kinases, tumorigenesis, cancer progression

## Abstract

In recent years, copper function has been expanded beyond its consolidated role as a cofactor of enzyme catalysis. Recent papers have demonstrated a new dynamic role for copper in the regulation of cell signaling pathways through direct interaction with protein kinases, modulating their activity. The activation of these pathways is exacerbated in cancer cells to sustain the different steps of tumor growth and dissemination. This review will focus on a novel proposed role for the transition metal copper as a regulator of cell signaling pathways through direct interaction with known protein kinases, which exhibit binding domains for this metal. Activation of these pathways in cancer cells supports both tumor growth and dissemination. In addition to the description of the results recently reported in the literature on the subject, relevance will be given to the possibility of controlling the cellular levels of copper and its homeostatic regulators. Overall, these findings may be of central relevance in order to propose copper and its homeostatic regulators as possible targets for novel therapies, which may act synergistically to those already existing to control cancer growth and dissemination.

## 1. Systemic and Cellular Copper Homeostasis

Copper (Cu) is a transition metal essential for the physiological growth and development of all living organisms. Due to its property of exchanging between reduced (CuI) and oxidized forms (CuII), it is involved in several cellular processes, acting as a catalytic cofactor for a multitude of enzymes named cuproenzymes [1], including both intracellular and secreted or membrane-embedded proteins, the latter receiving copper during their maturation. Some of these are enzymes involved in free radical detoxification, such as the Cu/Zn superoxide dismutase 1 and 3 (SOD1 and SOD3) that convert superoxide (O^2.-^) into molecular oxygen (O_2_) and hydrogen peroxide (H_2_O_2_), and in energy production, such as the cytochrome c oxidase that is involved in cellular respiration [2]. Moreover, enzymes related to iron homeostasis (i.e., ceruloplasmin and hephaestin) and others involved in the synthesis of melanin (i.e., tyrosinase) bind copper. Additionally, amine oxidases require copper to catalyze the oxidation of amine species (i.e., histamine) into aldehydes and ammonia. Among them, extracellular lysyl oxidase (LOX) and lysyl oxidase-like (LOXL) enzymes need copper to catalyze the oxidative deamination of lysine residues to cross-link components of the extracellular matrix (ECM), such as collagen and elastin. Copper is also fundamental in the physiology of the nervous system. Specifically, the dopamine β-hydroxylase catalyzes the last step in the synthesis of norepinephrine through a copper- and ascorbate-dependent hydroxylation [3,4].

As a trace element, in humans, copper is acquired from the diet with a recommended daily intake of 0.9 mg/day in healthy adults [5]. Among the foods of animal origin, particularly rich in copper are liver and fishery products, while small amounts of copper are contained in meat, eggs and milk. Among vegetables, dried fruit, whole grains and legumes are a good source of copper. A reasonable amount of copper is contained in cocoa powder and in mushrooms, while drinking water contains copper at very low quantities [6].

The adult body contains about 110–120 mg of copper, mainly concentrated in the kidneys, brain, heart, cerebrospinal fluid, hair, nails and liver [7]. Copper is first absorbed by enterocytes in the small intestine, transferred to serum albumin and conveyed by blood to the liver via the portal circulation [8]. The liver represents the major store of copper in the whole body [5,7]. Liver synthesizes the main copper-binding circulating protein ceruloplasmin, which is fundamental for oxidized iron transport by transferrin, since it is a ferro-oxidase. However, it has been demonstrated that ceruloplasmin does not deliver copper to cells [9].

In mammalian cells, the high-affinity transporter CTR1 (SLC31A1) located in the plasma membrane mainly mediates copper uptake. CTR1 belongs to the SLC31 family of copper permeases present in all eukaryotes; it is a trimeric protein, with a pore that allows for the inward flow of CuI. Each monomer is characterized by methionine rings created by an MXXXM motif conserved in the second transmembrane domain of all SLC31 family members, which is essential for the activity of human CTR1 [10]. The correct regulation of copper homeostasis is fundamental for appropriate organism development. A deletion of the CTR1 gene, related to a drastic drop of intracellular copper levels, produces severe developmental retard and morphological abnormalities in a mouse model [11]. Close to the plasma membrane, in mammals, the STEAP family of ferric reductases (STEAP1-4) reduces CuII to CuI, rendering it available for cellular intake by CTR1 [12,13]. Cells can regulate copper homeostasis in response to copper availability, modulating both the mRNA and the cellular localization of CTR1. Upon copper-depleted conditions, CTR1 gene is transcriptionally induced by the zinc-finger transcription factor Specificity Protein 1 (Sp1) [14]. By contrast, elevated copper concentrations stimulate CTR1 endocytosis to reduce copper intake [15]. The recycling of CTR1 from the plasma membrane to endosomes and the back-in response to alterations of copper homeostasis is mediated either by the retromer, a heterotrimeric protein complex of VPS35, VPS29 and VPS26 [16] or by its proteasome-induced degradation [15]. Moreover, it was demonstrated that a protein homologous of CTR1, namely CTR2, can cleave CTR1 ectodomain (tCTR1) reducing its copper import activity [17]. tCTR1 seems to be fundamental in the mobilization of endosomal copper pools, making it available for export out of the cells [18].

Due to its high redox reactivity as a free ion, unbound copper is extremely toxic for cells catalyzing the Fenton and Haber–Weiss reactions with the hydrogen peroxide (H_2_O_2_) and the superoxide anions (O^2.-^), respectively, leading to the production of the hydroxyl radical (OH^.^) [19]. These reactive oxygen species (ROS) can damage lipids, proteins, and nucleic acids, leading to cell death [20]. For these reasons, free copper is maintained at extremely low levels (10^−15^–10^−21^ M) and is mainly bound to sentinel proteins with metal binding domains, such as those belonging to the metallothionein family (MT1-4) [21] and to the tripeptide glutathione (GSH) [22]. MTs are cysteine-rich metal-binding proteins with high affinity for several essential and toxic divalent and monovalent transition metals. MTs protect cells against copper toxicity by forming irreversible metal–thiolate clusters within their structure [23]. Conversely, GSH not only prevents the presence of free copper ions but can ferry it to higher affinity proteins known as metallochaperones that have conserved copper-binding motifs (MTCXXC) [24].

**Figure 1:**
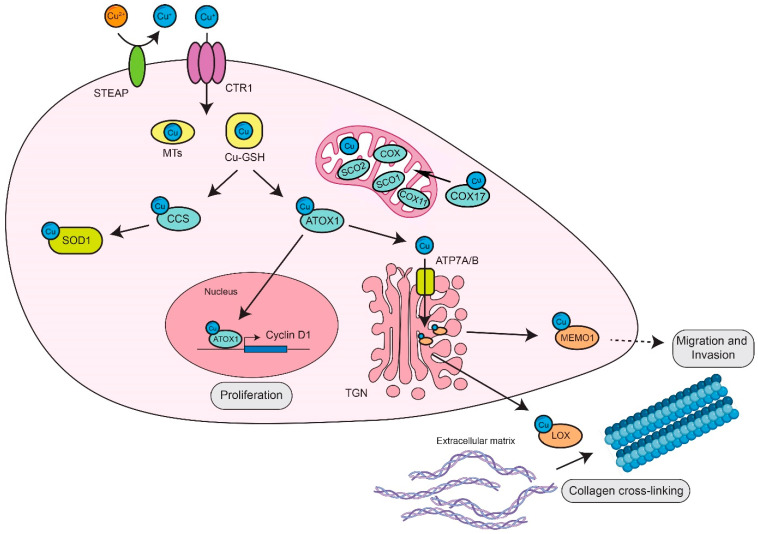
The main routes of copper distribution in cells.

Copper chaperones orchestrate copper homeostasis inside cells by both preventing noxious copper reactions and by delivering copper to cuproenzymes either during their biosynthesis or soon after. They are present in all subcellular sites (cytosol, mitochondria, endoplasmic reticulum, trans-Golgi network) and have preferential cuproprotein targets (Figure 1).

Cytosolic CCS (copper chaperone for superoxide dismutase) delivers copper into the active site of the cytosolic SOD1 [25]. CCS is of focal utility to monitor copper level in cells.

CCS represents a sensor of intracellular copper levels since its expression is modulated by intracellular copper status. Bertinato and colleagues demonstrated in vivo a dose-dependent upregulation of CCS in tissues of rats fed with low copper diets. Additionally, in vitro analyses from the same group demonstrated that switching cells from copper-depleted to copper-overloaded medium rapidly promoted CCS degradation by the proteasome [26,27].

Another cytosolic copper chaperone, ATOX1 (Antioxidant-protein 1, also known as HAH1), ferries copper to the P-type ATPases ATP7A and ATP7B located in the trans-Golgi network (TGN). Specifically, ATP7A and B are P-type ATPases membrane pumps with a common molecular structure that includes in their cytoplasmic N-terminal region six metal-binding sites (MXCXXC) and an intramembranous copper-binding CPC motif [28]. ATP7A and ATP7B play two distinct roles inside the cells: the first one, in the TGN, is to supply copper to newly synthetized cuproenzymes to be secreted from cells (e.g., ceruloplasmin and LOX/LOXL proteins) and the second one is at the plasma membrane where these pumps promote the excretion of excessive copper, to preserve intracellular copper levels. ATP7A and B are highly homologous [29] even if they show a different pattern of expression in different cell types (Figure 1). ATP7A is expressed in most tissues, especially in intestinal enterocytes, ensuring dietary copper absorption [30], whereas ATP7B is primarily present in the liver, in which it favors copper excretion into the bile, but also in the kidneys and to a lesser extent in the brain, heart and lungs [31]. In response to elevated copper levels, ATP7A and ATP7B are re-localized into vesicles that move toward the basolateral and apical membrane, respectively, although with different paths of migration. Upon the restoration of physiological Cu levels, ATP7A/B return to the TGN through endosomal compartments, processes orchestrated by several protein complexes including adaptor complex AP-1 and AP-2, retromer, Arp2/3, WASH, BLOC-1 and COMMD/CCDC22/CCDC93 complexes [32].

Mutations in ATP7A/B pumps determine alterations in copper metabolism. Specifically, loss of function mutations in the ATP7A gene cause Menkes’ disease, a rare X-linked inherited disorder that affects males in pediatric age. Reduced ATP7A-dependent copper export from intestinal enterocytes into the plasma is responsible for the systemic deficiency of copper, which occurs in affected individuals. Copper is particularly low in the brain where ATP7A activity is necessary for transport through the blood–brain barrier. The impairment of cuproenzymes activities, lacking their cofactor, results in the peculiar phenotype of the affected patients, who show altered collagen synthesis and alteration of the structure of blood vessels, hair hypopigmentation, and hypothermia. It is a fatal disease, mainly due to neurodegeneration, for which a definitive cure is far to be found. Xiao and colleagues confirmed a crucial connection between copper homeostasis and hormone biosynthesis, demonstrating that the alteration of copper homeostasis achieved by a genetic mutation of the Cu exporter ATP7A produces a reduction in norepinephrine synthesis and a dysregulation of rest–activity cycles and arousal [33]. Mutations in ATP7B gene give rise to Wilson’s disease, in which defects in the ATP7B-mediated biliary copper excretion cause hepatic and systemic copper overload [34]. Necrosis of hepatocytes releases excess copper into the circulation and creates copper overload in other organs. Typical is the formation of copper deposition in the cornea, producing Keyser Flesher rings, representing hallmarks of the malady. Furthermore, excess copper reaches the cerebral cortex, basal ganglia, white matter, and thalamus, resulting in neurological alterations. 

Both Wilson’s and Menkes disease are characterized by low levels of blood ceruloplasmin, in that copper is available but mutated ATP7B cannot deliver it to the nascent protein, and copper is lacking systemically, because it does not pass the enterocytes. Wilson’ disease is not life-threatening in most cases. The excess copper can be controlled and excreted by treatment by copper-chelating agents and well-established drugs such as D-penicillamine, tetrathiomolibdate (TTM) or triethylene tetramine (TEPA or TRIEN) [35].

Mitochondrial copper entrance and distribution have been the subjects of study for several years. The importance of mitochondrial copper homeostasis resides in the fact that the most relevant cuproenzyme in these organelles, cytochrome c oxidase (COX), a multi-subunit protein, represents the complex IV of the respiratory chain, and it is essential for respiration and ATP production [2]. The cytochrome c oxidase copper chaperone (COX17), together with other mitochondrial proteins (e.g., SCO1, SCO2 and COX11), provides copper to COX [2]. Specifically, in subunit I and II, it shows two iron centers (heme a and a3) and two copper centers (a binuclear copper center, CuA, and CuB) performing catalytic activity, which confers to this enzyme the capability to reduce O_2_ to H_2_O, and to contribute to the formation of mitochondrial ΔΨ, ultimately allowing for cell respiration and ATP synthesis (Figure 1).

The dysregulation of copper homeostasis results not only in genetic disorders but also in neurodegenerative diseases [36]. In Alzheimer’s disease, copper binds directly to Aβ peptides, promoting their aggregation, whereas in Parkinson’s disease, it interacts with α-synuclein, producing toxic oligomers [37,38]. These copper–protein aggregates induce oxidative stress and cell damage [39].

## 2. Role of Copper Homeostasis and Copper-Binding Proteins in Cancer

The disruption of copper homeostasis seems to be involved in multiple steps of cancer biology (Table 1).

Many studies have demonstrated that the copper demand of cancer cells is higher than in normal ones, as demonstrated by the increased copper levels observed in cancer patients’ serum and tumors [9]. Furthermore, Blockhuys and colleagues analyzed the RNA levels of 54 identified copper-binding proteins in different tumor tissues, demonstrating that many of them were differently expressed in cancer cells [62]. Cu-binding proteins seem to be implicated in cancer inception, growth and metastasis formation. For example, ATOX1 is involved in cell proliferation, by acting as a copper-dependent transcription factor that promotes cyclin D1 expression [66], and in the stimulation of inflammatory neovascularization [85].

Copper also promotes the ability of cancer cells to metastasize, through the activation of LOX/LOXL proteins and of the phosphotyrosine-binding protein MEMO1 (Mediator of ErbB2-driven cell motility). Specifically, LOX/LOXL proteins are secreted by cancer cells to increase collagen linearization and tissue stiffness, promoting metastatic dissemination, and to form a premetastatic niche in a distant site [86]. Instead, MEMO1 accumulates at the leading edge of cells where it interacts with IRS1 (insulin-like receptor substrate 1), activating the PI3K/AKT/Snail pathway, thus triggering the epithelial to mesenchymal transition (EMT) [87]. Additionally, copper is required for the activation of the transcription factor HIF-1 (Hypoxia Inducible Factor-1) by regulating the binding of HIF-1 α to the hypoxia responsive elements (HREs) on target genes involved in blood vessel development which allows for vascularization for tumor survival and EMT [88,89].

## 3. Copper-Dependent Kinases and Their Pathological Roles in Cancer

### 3.1. MEK Is a Copper-Binding Kinase That Triggers the RAS/RAF/MEK/ERK Pathway

**Figure 2:**
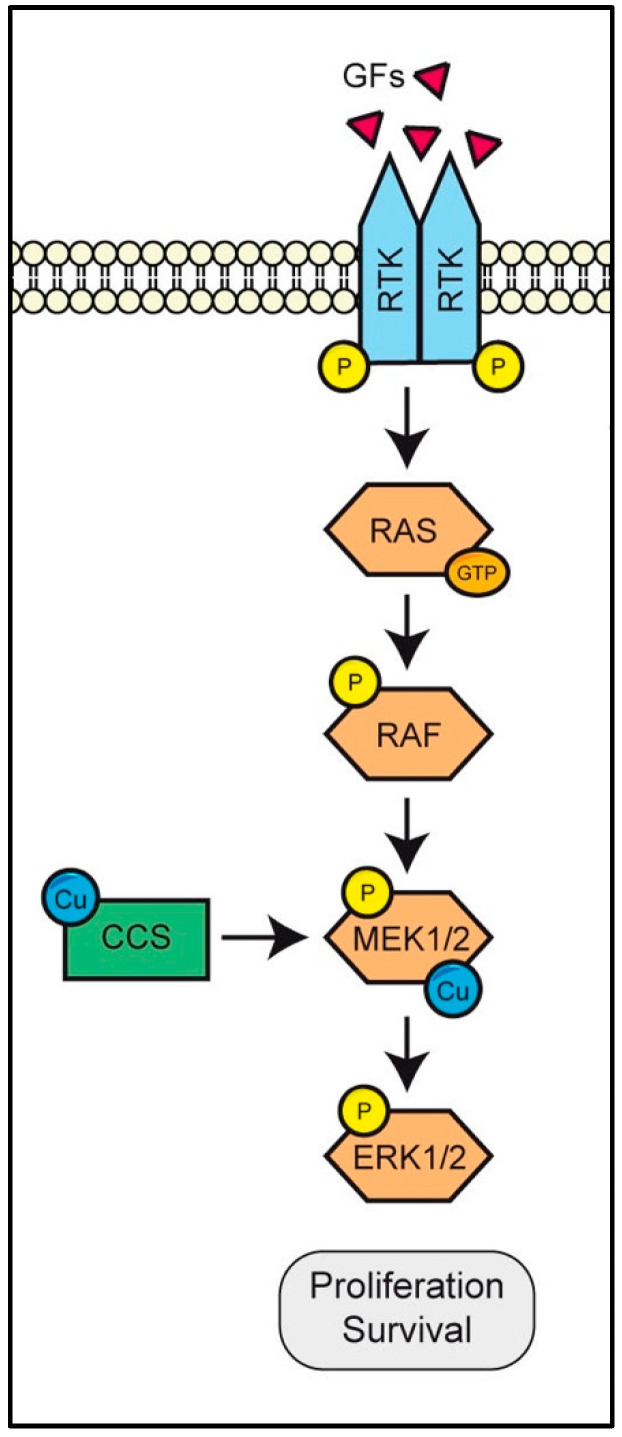
Copper modulates the RAS/RAF/MEK/ERK signaling axis. Upon engagement of receptor tyrosine kinase (RTK) by growth factors (GFs), following phosphorylation of RAS e RAF, the copper chaperone for superoxide dismutase 1 (CCS) delivers copper to the mitogen-activated protein kinases (MAPKs) MEK1/2. The binding of copper to MEK1/2 is necessary for physical interaction of MEK1/2 with ERK1/2 and, in turn, for ERK1/2 activation by phosphorylation.

Mitogen-activated protein kinase kinase 1/2 (MEK1/2) was the first-identified copper-binding kinase. It belongs to the RAS/RAF/MEK/ERK pathway, involved in many physiological (e.g., cell proliferation, survival, differentiation, motility and metabolism [90]) and pathological (e.g., cancer initiation and progression [91]) processes (Figure 2).

The RAS/RAF/MEK/ERK pathway responds to a wide variety of extracellular stimuli prompting various receptors, such as the receptor tyrosine kinases (RTK). In most cases, the activation of such receptors results in the activation of the small GTPase RAS. Upon cellular stimulation, through the action of GTPases-activating proteins (GAPs), RAS switches between an inactive GDP-binding conformation to an active GTP-binding conformation. There are three gene isoforms of RAS (i.e., H-RAS, K-RAS and N-RAS), which encode for proteins with highly homologous sequences and quite different activities, tissue expression patterns and preferred targets.

The dysregulation of the RAS/RAF/MEK/ERK axis pathway is closely related to the occurrence and development of tumors [92]. Specifically, overexpression and activating mutations of RTK, as well as of components of this cascade, are extremely frequent in tumors. Oncogenic RAS mutations can be classified into two main groups: mutations on Gly12 or 13 (G12/13) that impair GAP associations and mutations on Gln61 (Q61) that decrease the intrinsic GTPase activity of RAS. Both types of mutations cause an extension of the half-life of GTP-loaded RAS, resulting in hyperactivation of the entire pathway. K-RAS is the most mutated isoform in all cancers (85%), followed by N-RAS (12%) and H-RAS (3%).

The pathway then proceeds with the activation of RAF (Figure 2). There are three RAF isoforms with different kinase activities: B-RAF, which is the most active isoform, followed by C-RAF and A-RAF. An activating mutation in B-RAF that converts Val 600 into Glu (V600E) has been observed in many types of tumors including melanomas [93] and thyroids cancers [94]. RAF molecular structure comprises three conserved regions (CR): CR1, CR2 and CR3. CR1, located at the amino terminal region, contains a Cys-rich domain and the RAS-binding domain (RBD); CR2, near the amino terminus, is characterized by a Ser-Thr rich sequence. CR3, located at the carboxyl terminus, is the catalytic functional region with an N-terminal acidic motif (NTA) and a C-terminal regulatory tail. RAF is another predominant target of oncogenic mutations. Once RAF is activated, its CR3 can interact with MEK, inducing its phosphorylation on Ser218 and Ser222 that spreads the pathway activation.

There are two isoforms of MEK (i.e., MEK1 and MEK2), which have a short regulatory N-terminus and a canonic kinase domain. The N-terminal regulatory region of MEK1/2 contains a docking site for ERK substrate, a nuclear export sequence that controls the cytoplasmic–nuclear shuttling of the protein, and a negative regulatory sequence that forms a helix that locks the kinase in an inactive status (Figure 2). MEK kinases are dual-specificity kinases that activate the Ser/Thr protein kinase ERK, by phosphorylation of both Tyr204 and Thr202 residues. This recognition and activation mechanism confers double specificity and improves the accuracy of the signal transduction, preventing errors in ERK activation. The ERK family is composed of five members (i.e., ERK 1, 2, 3, 5 and 6) of which only ERK1 and ERK2 are involved in the RAS/RAF/MEK/ERK pathway [95]. To achieve its function, ERK needs to translocate into the nucleus, and MEK favors this process. When the signaling pathway is shot down, ERK is retained in the cytoplasm by specific scaffold proteins (e.g., PEA-15, Sef1, and hScrib) [96], whereas upon cellular stimulation, MEK-mediated phosphorylation leads the detachment of ERK from the anchors and its nuclear import, where it regulates the activity of hundreds of transcription factors [97]. Among the targets of ERK, some are implicated in cell proliferation such as c-Fos [98], c-Jun [99] and c-Myc [100], and others promote cell migration by cytoskeletal remodeling such as Abi1 [101] that promotes actin filaments polymerization and MLCK [102] in lamellipodia extension. Cytoplasmic ERK can modulate the activation of the entire pathway by phosphorylating the upstream protein kinases in a negative feedback loop [103].

The most common mutations regard the *RAS* gene (mainly that coding for the isoforms K-RAS), involving around 30% of all cancer types; mutations in the *RAF* gene are around 8% of all cancer types, whereas mutations in the MEK and ERK kinases are less frequent (1%). It was demonstrated that inhibition of this pathway can reprogram cancer cells to a non-transformed phenotype in vitro and can inhibit tumor growth in vivo [104].

The first evidence about the role of copper as a positive allosteric regulator of kinases activity dates back to 2012. Tursky and colleagues [67] demonstrated, through an in vitro biochemical analysis using purified recombinant MEK1, that this kinase binds two CuII atoms with high affinity (K_D_ 10^−18^ M) and that its ability to phosphorylate ERK is enhanced by copper in a dose-dependent manner (Figure 2). In particular, the knockdown of the gene coding for the high copper affinity transporter CTR1 in flies and mouse cells, or the use of copper chelators such as tetrathiomolybdate (TTM) and bathocuproine disulfonate (BCS), impaired the phosphorylation of ERK promoted by MEK1. Moreover, they found that intracellular copper adequate conditions are fundamental to achieve ERK1 activation, because copper is required to stimulate the MEK1-ERK1 physical interaction that precedes ERK phosphorylation by MEK1. This was further confirmed by the findings of Brady et al. who demonstrated that MEK1 mutation in its putative copper-binding site, rich in histidine and methionine residues (H188, M187, M230, and H239), resulted in its inactivity [74].

These findings, supporting a new role of copper as a dynamic signal metal required for the modulation of kinases by binding to allosteric sites, lead in 2015 to the introduction of the term “metalloallostery” by Chang and colleagues [105]. Then, the focus moved to the research of the copper chaperone in charge of delivering the metal to MEK1. Grasso and colleagues recently demonstrated, by using surface plasmon resonance and proximity-dependent biotin ligase analyses, which CCS is the copper donor appointee of the copper transfer to MEK1 [69]. They further corroborated their findings by using a CCS small-molecule inhibitor or by introducing mutations in the CuI binding site of CCS. Both approaches resulted in an impairment of MEK1 activity. However, the process of copper delivery to MEK from CCS is still elusive [69].

These intriguing discoveries inspired researchers to analyze its possible implication in the modulation of kinase pathways deeply involved in tumorigenesis. This would also justify the higher level of copper found in the serum of cancer patients [52] (the higher demand of copper in cancer cells compared to healthy ones [106]) and the anti-metastatic potential of tumor treatments with copper chelators [107].

Further advances on the ability of copper to function as an allosteric regulator of the RAS-ERK axis and demonstrating the involvement of well-known copper chaperones in this mechanism were not long in coming. In 2019, Kim and colleagues found that the copper chaperone ATOX1, highly expressed in melanoma cells, underlies the BRAF^V600E^ signaling cascade [79] (Figure 2). A work published in 2017 by Blockhuys et al. [62] already consolidated the idea that copper-binding proteins played a key role in tumorigenesis by showing the modulation of the human copper proteome in several cancers. In particular, by analyzing the expression levels of known copper-binding proteins (including transporters, chaperones, pumps and enzymes) in different cancers, using the available database resources of RNA transcript levels, they found that many of the copper-binding proteins were up- or downregulated. Moreover, they demonstrated the overexpression of the ATOX1 chaperone in breast cancer [62]. Following, Kim et al. [79] demonstrated both the upregulation of this chaperone in melanoma and its correlation with poor survival in melanoma patients, due to the role of ATOX1 in the stimulation of BRAF^V600E^ melanoma growth [79]. The authors showed that the knockout of *ATOX* decreased ERK1/2 phosphorylation and reduced cell growth in A375 and WM888 melanoma cell lines harboring the oncogenic mutations in the Ser/Thr kinase BRAF. Additionally, the pharmacological inhibition of ATOX1, using the inhibitor DCAC50 [108], which also blocks copper transfer to other cuproenzymes, reduced cell proliferation in a panel of cell lines including human lung cancer, leukemia cancer and breast cancer cell lines with minimal effects in the parental cells [108].

### 3.2. ULK1/2 Is A Copper-Binding Kinase Essential for Autophagy Initiation

**Figure 3:**
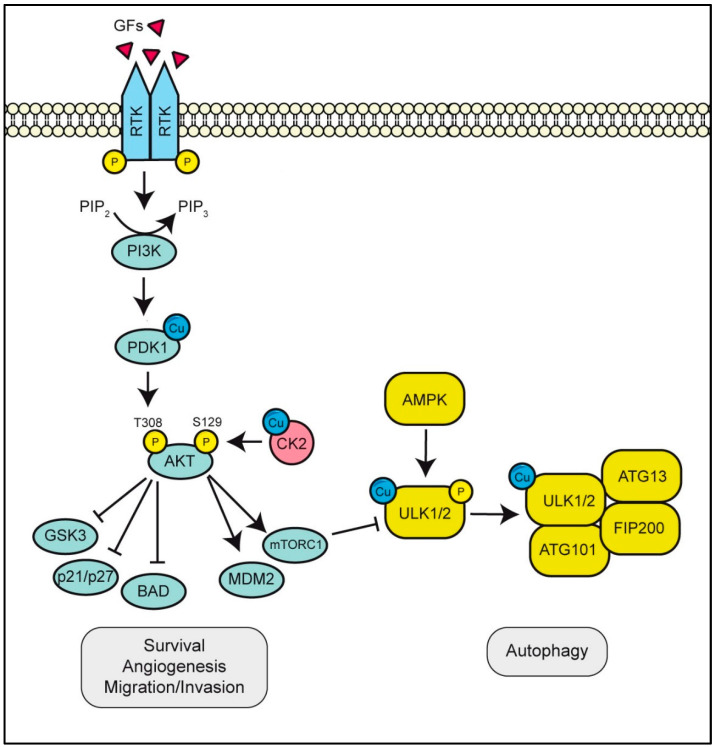
Copper functions as a positive allosteric regulator of the PI3K/AKT signaling cascade and of the autophagic proteins Unc-51-like kinase 1 and 2 (ULK1 and ULK2). Upon engagement of receptor tyrosine kinase (RTK) by growth factors (GFs), phosphoinositide 3-kinases (PI3K) phosphorylate phosphatidylinositol-4,5-bisphosphate (PIP2) to generate phosphatidylinositol-3,4,5-trisphosphate (PIP3), recruiting the Ser and Thr kinase AKT. The production of PIP3 by PI3Ks recruits the copper-dependent PDK1, which in turn phosphorylates AKT on its Thr308. The phosphorylation of AKT at the Ser129 residue proceeds through the copper-dependent casein kinase 2 (CK2). Overall, the activation of the PI3K/AKT pathway confers to cancer cells a more aggressive behavior. Furthermore, the binding of copper is required for the kinase activity of ULK1/2, leading to the formation of a functional protein complex, together with mATG13, FIP200 and ATG101 to regulate the initiation of autophagy.

The discovery that copper works as a modulator of MEK1 activity raised the hypothesis that this metal could be required for the activation of other kinases. Recently, Tsang and collaborators [80] described the copper dependence of the autophagic flow: an increased intracellular bioavailability of copper was necessary for the starvation-induced autophagy and the formation of the autophagosome complex, whereas the CRISPR-mediated ablation of the *CTR1* gene, and thus the reduction of intracellular copper content, led to an impairment of autophagy [80]. Of note, upon alignment of the MEK1 copper-binding sequence against other kinase domains, they identified the same copper-binding amino acid sequence found in MEK1 (i.e., His136, Met188 and His197) in the autophagic proteins Unc-51-like kinase 1 and 2 (ULK1 and ULK2) [80] (Figure 3). ULK is a cytoplasmic kinase that plays a pivotal role in the early steps of autophagy. Its gene is homologue to the *ATG1* gene that in the mammalian genome contains five *ATG1* homologues: *ULK1, ULK2, ULK3, ULK4* and *STK36*. Among them, only ULK1 and ULK2 show extensive similarity of sequence over the entire protein length, whereas ULK3, ULK4 and STRK36 have similarity only in the kinase structure domain. All these proteins have an N-terminal Ser/Thr protein kinase domain (KD) and a C-terminal interacting domain (CTD). A Ser/Pro-rich region is the main site of post-translational modifications. ULK1 forms a protein complex together with mATG13, FIP200, and ATG101 to regulate the initiation of autophagy.

The role of ULK1/2 and autophagy in cancer biology is controversial and depends on the specific tumor type and the stage of the disease. In the early stages of tumorigenesis, autophagy exerts an anti-tumorigenic role by killing cancer cells. By contrast, when the tumor is formed, in response to different types of stress, the survival and aggressiveness of tumor cells can increase [109]. Considering the dual role of autophagy, differential strategies have been employed using ULK1 inhibitors or activators depending on the cancer type. For instance, it has been demonstrated that low expression of ULK1 in operable breast cancer tissues is a marker of poor prognosis [110]. Therefore, in the case of breast cancer, the activation of ULK1 can be used as an effective anti-cancer strategy. Ouyang and colleagues identified a small molecule activator of ULK1, LYN1604, which regulated ULK1 and interacted with protein ATF3, RAD21 and *CASP3*/caspase3 to induce autophagy-related cell death in triple-negative breast cancer cells [111]. Additionally, many studies highlighted the potent chemotherapeutic effect of compounds derived from natural products. It was demonstrated that an aqueous extract of clove can inhibit tumor growth by activating the AMPK/ULK1 pathway [112]. Alongside ULK1 activators, Martin and colleagues identified two ULK1 small-molecule inhibitors, ULK-100 and ULK-101, which can disrupt autophagy, making tumor cells more sensitive to nutrient depletion [113].

Mutations of the amino acids contained in the putative Cu-binding sequence of ULK1/2 (His188, Met230 and His239) in Ala resulted in the reduction of the kinase activity without affecting its three-dimensional structure since the mutant was able to maintain its interactions with canonical targets as ATG13, ATG101 and FIP200. Thus, by in vitro assays using increasing concentrations of CuCl_2_, the authors demonstrated that Cu(I) promotes ULK1 and ULK2 activity in a dose-dependent manner [80]. Hence, the function of copper as a modulator of autophagy relies in its ability to positively modulate the activity of ULK1. As confirmed, in embryonic fibroblasts of *ULK1* and *ULK2* knockout mice, the authors observed that the expression of wild-type ULK allowed for the autophagic flow while the expression of ULK harboring mutations in the identified copper binding domain was unable to prompt autophagy. This work is particularly relevant because it establishes a link between copper ability to modulate autophagy and the KRAS pathway: in a mouse model of *Kras*^G12D^-driven lung cancer, the binding of copper to ULK1 is mandatory for autophagy and to support tumorigenesis in KRAS [64].

In the context of cancer biology, the discovery of another kinase, modulated by copper and deeply involved in tumorigenesis, led to a further development of this topic in copper-related research. The same research group more recently validated the hypothesis by demonstrating that in mouse BRAF^V600E^-driven lung adenocarcinoma, the reduction of intracellular copper content, achieved by *CTR1* deletion or by the use of the copper chelator TTM, simultaneously affected autophagic and MAPK signaling, thus leading to the reduction of lung adenocarcinoma growth [114].

### 3.3. PDK1 or CK2 Binds Cu Activating the PI3K/AKT Pathway

Tursky and collaborators [67], during their studies on copper involvement in the modulation of ERK1/2 signaling, found that CTR1 and copper-responsive components of RAS signaling lie downstream of RAS and do not impact the RAS/PI3K (phosphoinositide 3-kinases)/AKT signaling cascade [67]. Nine years later, Guo and colleagues [115] questioned these findings. They discovered that copper, on the contrary, is an important multilevel modulator of the PI3K/AKT network. Specifically, these authors demonstrated that phosphoinositide-dependent kinase-1 (PDK1) is a copper-binding kinase [115] (Figure 3). The binding of copper is required for the stimulation of PDK1 interaction with AKT and in turn its subsequent phosphorylation at the Thr308 residue. The authors identified different putative copper-binding sites in PDK1 kinase domain through the study on copper-induced in vitro oxidative stress, coupled to mass spectrometry, and found that copper binding also prompted the activation of PDK1 [115]. These authors also demonstrated that PDK1 binds copper mainly to His117 and His203 residues. Mutation of these amino acids to Ala (and to a lesser extent of Met134 and His197) impaired the binding of copper to PDK1, a condition that is worsened in the double mutant His117Ala/His203Ala [115]. The reduction of copper–PDK1 interaction disrupted PDK1 activity, as demonstrated by a decrease in AKT phosphorylation at Thr308. To demonstrate that the activation of the PI3K/AKT pathway relies on intracellular copper content, the authors tested other ions, and none of them enhanced the interaction between PDK1 and AKT. Furthermore, PI3K inhibitors, such as LY294002 and wortmannin, could not abrogate the binding of copper to PDK1, indicating that copper binds PDK1 independently of PIP3-mediated PDK1 plasma membrane translocation [115].

The PI3K/AKT signaling pathway is one of the major pathways dysregulated in cancer [116]. It is involved in the control of several cancer-related processes such as metastasis formation, metabolic rewiring, angiogenesis and inflammation [117]. The induction of this pathway is mediated by the binding of growth factors to different receptors, such as RTK family, TLRs (Toll-like receptors) and BCRs (B-cell antigen receptors). Upon receptor activation, PI3Ks phosphorylate phosphatidylinositol-4,5-bisphosphate (PIP2) to generate phosphatidylinositol-3,4,5-trisphosphate (PIP3) that recruits, near the plasma membrane, oncogenic signaling proteins including the Ser and Thr kinase AKT [118]. The production of PIP3 by PI3Ks recruits PDK1 to the plasma membrane, which in turn phosphorylates AKT in its Thr308. AKT becomes fully activated after an additional phosphorylation on Ser473 by mTORC2, also known as PDK2. AKT is also activated by IGF1, the binding of which IGF1R recruits and activates IRS1 and PI3K.

AKT dysfunctions are correlated to several diseases, including cancer [119]. Many effectors modulated by AKT are important in tumor growth, tumor survival, cancer immunity, cancer metabolism and angiogenesis [120]. It is not surprising that the PI3K/AKT pathway is upregulated in many tumors. AKT phosphorylates cyclin-dependent kinase inhibitors and prevents p27 translocation to the nucleus, which in physiological conditions inhibits the cell cycle [121]. AKT inhibits the proapoptotic factors Bad and procaspase-9 through their phosphorylation, and Bcl-2 homology domain 3-only proteins, increasing the survival rate of cancer cells.

The dysregulation of PI3K/AKT signaling also plays an important role in cancer drug resistance. A study demonstrated that the IGF1R/p110β/AKT/mTOR axis confers resistance to BYL-719 in PIK3CA mutant breast cancers [122]. PI3K/AKT signaling is involved in metabolic rewiring, essential to supporting the anabolic needs of cancer cells. AKT regulates metabolism-associated proteins such as Sterol Regulatory Element-Binding Protein (SREBP), activates mTORC1 that enhances glucose metabolism and lipid synthesis, and inhibits glycogen synthase kinase 3 (GSK3) that suppresses the glycogen synthase [117]. The PI3K/AKT pathway promotes cancer metastatization by reducing intercellular adhesion, remodeling the cytoskeleton and inducing EMT [123].

At the same time, Chojnowski and colleagues [84] investigated if CK2 (also called casein kinase 2 or CKII) could be a putative copper-dependent kinase, using the same strategy of the research groups that demonstrate the presence of copper-binding motifs in ULK1/2 and in PDK1 [84]. Among other functions, CK2 potentiates oncogenic signaling such as the PI3K/AKT pathway, by phosphorylating AKT at Ser129, thus finalizing its activation through the stabilization of the PDK1-depentent phosphorylation at Thr308 [84] (Figure 3). By analyzing the homology sequence between CK2α and MEK1, two residues (Met153 and His154) were pinpointed as copper-binding sites. CK2 is a Ser/Thr kinase that has hundreds of physiological substrates, and for this reason, it is responsible for the generation of over the 20% of the human phosphoproteome [84]. Mammalian CK2 is a tetrameric enzyme composed of two catalytic α subunits and two regulatory β subunits. CK2 prompts tumorigenesis and metastatization, favoring cell survival, by directly interfering with caspases action or by activating the caspases inhibitor ARC and supporting neovascularization [124]. Furthermore, it contributes to multi-drug resistance by enhancing the expression of the extrusion pumps P-gp, MRP1 and BCRP favoring drug efflux from cells. Moreover, CK2 acts on the IKK (IkB) kinase/NFkB pathway, favoring IkBα (inhibitor of NFkB) degradation by direct phosphorylation or through the activation of IKK with the consequent release of NFkB and its nuclear translocation [125]. CK2 also targets the JAK2/STAT3 pathway, by phosphorylating both JAK2 and STAT3 proteins producing the abnormal amplification of cytokine signals [126].

To further investigate the putative interaction between copper and CK2, Chojnowski et al. performed an in silico analysis that revealed the presence of two copper-binding sites: one at protein surface and the other in the catalytic pocket [84]. The authors demonstrated that copper increased CK2 kinase activity as revealed by increased phosphorylation of Jabba, a CK2 substrate. By contrast, the CK2 mutant, in which the two putative copper-binding amino acids (Met153 and His154) were mutated to Ala, was no longer able to bind copper and to phosphorylate Jabba. Additionally, an ELISA assay demonstrated that the effect of copper on CK2 activity is nucleotide specific. Even if CK2 activity requires ATP or GTP, the enhancement of CK2 activity mediated by copper was observed only in the presence of ATP. Furthermore, in *CTR1*^-/-^ MEFs, there was observed a reduction in the phosphorylation of two CK2 substrates, AKT at Ser129 and CDC37 at Ser13. Finally, to demonstrate that CK2 activity depends on copper availability, the authors used a copper ionophore, namely Cu-ATSM (diacetylbis(4-methyl-3-thiosemicarbazonato)copper(II), known to facilitate copper delivery inside cells [84], and they observed that copper–ATSM was able to enhance CK2 activity, increasing the phosphorylation of its targets.

Thus, these last findings revealed a sophisticated regulation on the PI3K/AKT axis by copper. Copper modulates both the phosphorylation of AKT at the Thr308 residue (70% responsible for AKT activation) and the phosphorylation at the Ser129 residue of AKT, responsible for the accomplishment of the signaling cascade.

### 3.4. PDE3B Requires Copper to Modulate cAMP/PKA Pathway

**Figure 4:**
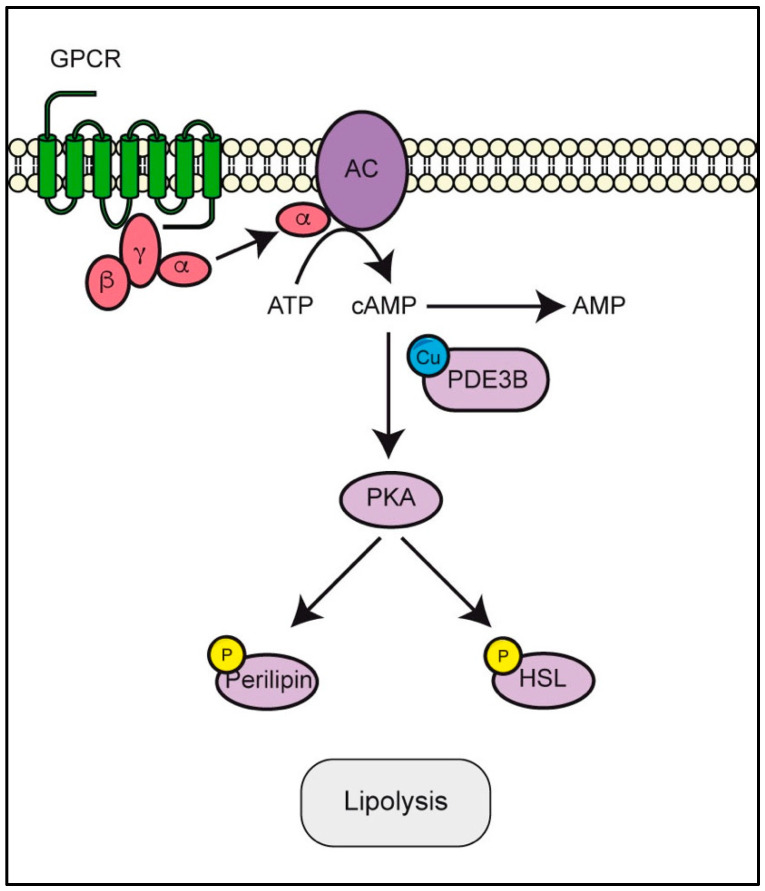
Copper modulates cyclic adenosine monophosphate (cAMP) intracellular levels. Upon the binding of extracellular stimuli to G-protein-coupled receptors (GPCRs), the Gα subunit is separated from the others (i.e., Gβ and Gγ) and activates adenylyl cyclases (ACs), leading to the conversion of ATP into cAMP. Copper inhibits phosphodiesterase 3B (PDE3B) through the binding to its Cys768 and Cys777 residues, resulting in the indirect regulation of the activity of protein kinase A (PKA), which in turn phosphorylates the hormone-sensitive lipase (HSL) and perilipin with the subsequent rewiring of lipid metabolism.

Copper seems to be involved in the regulation of cyclic adenosine monophosphate (cAMP) intracellular levels. Specifically, Krishnamoorthy and colleagues [127] demonstrated that copper directly binds and inhibits the cAMP-degrading phosphodiesterase, PDE3B [127] (Figure 4). Krishnamoorthy and colleagues demonstrated that PDE3B binds copper at two cysteines, Cys768 and Cys777, in its catalytic domain. Specifically, the authors illustrated a copper-dependent inhibition of PDE3B activity that was prevented using the copper-chelator BCS. Additionally, they highlighted the role of copper in amplifying the cAMP signal as demonstrated by the increment of cAMP levels and the increased phosphorylation of HSL and perilipin upon CuCl_2_ treatment [127]. cAMP is used by cells as a second messenger that can regulate several biological processes including cell growth, cell metabolism, differentiation and apoptosis [128]. Upon the binding of extracellular stimuli to G-protein coupled receptors (GPCRs), the Gα subunit is separated from the others (i.e., Gβ and Gγ) and activates adenylyl cyclases (ACs), leading to the conversion of ATP into cAMP. The concentration of cAMP is finely regulated inside cells by the balance between its generation, mediated by ACs activity, and its degradation, controlled by phosphodiesterases (PDEs).

Protein kinase A (PKA) represents the main target of cAMP that, once activated, phosphorylates Ser and Thr residues of downstream proteins. The cAMP/PKA pathway is frequently dysregulated in cancer cells in which it was demonstrated that PKA promotes cancer cell invasion and metastasis [129,130]. PKA is involved in cytoskeleton remodeling, by targeting both structural (e.g., actin, integrins and myosin light chain) and regulatory proteins (e.g., Rho GTPases and Src kinases) [131]. PKA also phosphorylates CDC24 interacting protein 4 (CIP4) and the focal adhesion kinase (FAK), and it favors the reprogramming of lipid metabolism by phosphorylation of the hormone-sensitive lipase (HSL) [132] and perilipin [133].

Thus, the modulation of cAMP level triggered by copper via PDE3B affects PKA, adding another kinase target of this transition metal.

## 4. Conclusions

Redox transition metals have always been recognized to play a paramount role in cells and organisms, as cofactors of enzymes involved in crucial steps of metabolism. First, both copper and iron participate in respiration and cell energy procurement, essential for organisms’ survival. Furthermore, they are involved in an array of other specific functions, which allow for pursuing many other accomplishments, ranging from the construction of the extracellular matrix, the adaptation to low oxygen levels, the synthesis of neurotransmitters and pigments, to the defense against reactive oxygen species.

However, this was only the beginning of a plethora of studies that have led to ever-new discoveries on the multiple roles that these metals play in organisms, leading to the finding that not only do they have a useful function, but that they also can be harmful if not strictly controlled. The literature on these aspects is now boundless, and possibly not entirely comprehensive, and it describes a complex network of protein transporters, chaperones, and regulators, which consent to the homeostasis of these metals, participating with both their delivery to the metabolic enzymes and the control over their possible noxious effects.

In time, and in particular, the disruption of copper homeostasis was demonstrated to be responsible for genetic diseases, either leading to metal depletion or overload, but also to neurodegenerative diseases (Alzheimer’s disease, Parkinson’s disease) in which metals may participate in aberrant protein fibrillation and oxidative stress damage. More recently, the involvement of transition metals in cancer onset, development and spreading has been called into question, offering novel ideas for the proposal of innovative therapeutic approaches. Iron-dependent cell proliferation (ferroplasia) relies on the regulation of signaling pathways and the opportunity of cancer treatment based on the induction of ferroptosis, a controlled way to eliminate malignant cells, which is repeatedly suggested [134,135].

Furthermore, a strong connection between copper and cancer came recently into light, leading to the definition of the term “cuproplasia”, a copper-dependent cell growth and proliferation [134]. This is not only simply because oxidative stress generated by dysregulated copper may induce cell transformation and uncontrolled proliferation (as the increased level of copper found in tumors of different origin or in the serum of cancer patients may suggest) but, more intriguingly, because this metal was found to bind and regulate the function of protein kinases, acting as positive or negative allosteric regulators. This might explain the higher requirement of copper levels by tumor cells in comparison with healthy cells. Upon the dysregulation of kinase function, they are engaged in cancer initiation, progression, and spreading and in the acquisition of drug resistance.

These results open up a vast scenario, linking the complex world of copper homeostasis to that of protein kinase regulation, which will pave the way for intense research activity and will open the prospect to the proposal of new therapeutic approaches. As described in the present review, consistent evidence exists on the connection between copper and several protein kinases: MEK, triggering the RAS/RAF/MEK/ERK pathway, closely related to the occurrence and development of tumors; ULK, playing a pivotal role in the early steps of autophagy; PDK1 or CK2, which activate the PI3K/AKT pathway; cAMP/PKA pathway, by means of PDE3B. This does not appear to be only one case; all these kinases harbor copper-binding domains, and the binding of the metal regulates their function.

Now, the challenge of the research in this field will be to understand how the different kinases acquire copper, which are the chaperones delivering them the metal and the underlying mechanisms, which are the culprits of the copper dysregulation in cancer patients. However, since we do know how to control copper excess and dysregulation by means of copper-chelating agents already used in the therapy of genetic copper disease characterized by overload of the metal, these consolidated molecules may be repurposed in chemotherapy, representing an immediate tool to control cancer growth and spread.

## Figures and Tables

**Table 1:** Major findings regarding the role of copper in tumorigenesis.

Evidence/Cuproproteins Involved	Year	Authors	Ref.
Copper is required for angiogenesis	1982; 1994; 1997; 2001; 2006	Raju, K.S. et al.; Lane, T.F. et al.; Soncin, F. et al.; Landriscina, M et al.; Juarez, J.G. et al.	[40]; [41]; [42]; [43]; [44]
Higher copper serum levels in cancer patients	1983, 1989	Margalioth, E.J., et al.; Coates, R.J. et al.	[45]; [46]
Elevated copper serum levels in lung cancer patients	1989, 1994, 2011	Diez, M. et al.; Oyama, T., et al. Jin, Y. et al.	[47]; [48]; [49]
Copper depletion reduced gliosarcoma growth in rat brains	1990	Brem, S.S. et al.	[50]; [51]
Phase I study: 90 days copper deficiency reduces cancer progression in 5 out of 6 patients with metastatic cancer	2000	Brewer, G.J. et al.	[52]
TTM treatment of orthotopic mouse model of head and neck squamous cell carcinoma reduces cancer growth and angiogenesis	2001	Cox, C. et al.	[53]
Elevated copper serum levels in breast cancer patients	2002, 2012, 2015, 2015, 2015	Kuo, H.W., et al.; Feng, J.F. et al.; Ding, X. et al.; Adeoti, M. et al.; Pavithra, V. et al.	[54]; [55]; [56]; [57]; [58]
Elevated copper serum levels in colorectal cancer patients	2003, 2017, 2018	Nayak, S.B., et al.; Stepien, M., et al., Sohrabi, M. et al.	[59]; [60]; [61]
Cuproproteins expression is elevated in human cancers	2017	Blockhyus, S. et al.	[62]
Elevated copper serum levels in oral and thyroid cancer patients	2006, 2017	Khanna, S.S. and Karjodkar, F.R.; Baltaci, A.K., et al.;	[63]; [64]
Elevated copper serum levels in stomach cancer patients	2007	Yaman, M., et al.	[65]
The copper chaperone ATOX1 modulates cell proliferation	2008	Itoh, S. et al.	[66]
Copper regulates the RAS/RAF/MEK/ERK signaling pathway	2012; 2020, 2021	Turski, M.L., et al.; Aubert, L. et al.; Grasso, M. et al.	[67]; [68]; [69]
The copper-dependent aminoxidase LOXL2 is required for epithelial to mesenchymal transition (EMT)	2013	Millanes-Romero, A. et al.	[70]
Elevated copper serum levels in gallbladder and prostate cancer patients	2014; 2013; 2020	Cai, H., et al.; Basu, S. et al.; Saleh, S.A.H. et al.	[71]; [72]; [73]
Copper modulate BRAF signaling	2014	Donita, B.C. et al.	[74]
MEMO1 is copper-dependent protein exerting a crucial role during EMT and metastatization	2014	MacDonald, G., et al.	[75]
Copper chelation blocks the growth of BRAF^V600E^ melanoma	2017	Brady, D.C., et al.	[76]
ATP7A transfers copper to the cuproenzyme LOXL2 and it is involved in tumorigenesis	2019	Shanbagh, V. et al.	[77]
Copper activates RTK signaling	2019	He, F. et al.	[78]
ATOX1 modulates MAPK signaling In BRAFV^600E^	2019	Kim, Y.J., et al.	[79]
Copper modulates ULK1/2 mediated autophagy in lung carcinoma	2020	Tsang, T., et al.	[80]
Copper affects PDL-1 expression	2020	Voli, F., et al.	[81]
Mitochondrial copper depletion impairs triple negative breast cancer growth in a mouse model.	2020	Cui, L., et al.	[82]
Copper modulates AKT cascade	2021, 2022	Guo, J. et al.; Chojnowski, J.E., et al.	[83]; [84]

## Data Availability

Not applicable.

## Abbreviations

AP-1/2	adaptor complex
ATOX1/HAH1	Antioxidant-protein 1
ATP7A/B	P-type ATPases
BCS	bathocuproine disulfonate
cAMP	cyclic adenosine monophosphate
CCS	copper chaperone for superoxide dismutase
CIP4	CDC24 interacting protein 4
CK2	casein kinase 2
COX	cytochrome c oxidase
COX17	cytochrome c oxidase copper chaperone
CR	conserved regions
CTD	C-terminal interacting domain
ECM	extracellular matrix
EMT	epithelial to mesenchymal transition
FAK	focal adhesion kinase
GAP	GTPases-activating proteins
GPCRs	G-protein coupled receptors
GSH	tripeptide glutathione
GSK3	glycogen synthase kinase 3
HIF-1	hypoxia inducible factor-1
HREs	hypoxia responsive elements
IRS1	insulin-like receptor substrate 1
KD	kinase domain
LOX	lysyl oxidase
LOXL	lysyl oxidase-like
MEK1/2	mitogen-activated protein kinase kinase 1/2
MEMO1	mediator of ErbB2-driven cell motility
MT1-4	metallothionein family
NTA	N-terminal acidic motif
PDK1	phosphoinositide-dependent kinase-1
PI3K	phosphoinositide 3-kinases
PIP2	phosphatidylinositol-4,5-bisphosphate
PIP3	phosphatidylinositol-3,4,5-trisphosphate
PKA	protein kinase A
RAS	rat sarcoma
RBD	RAS-binding domain
RTK	receptor tyrosine kinases
SOD1/3	Cu/Zn superoxide dismutase 1 and 3
Sp1	zinc-finger transcription factor Specificity Protein 1
TEPA or TRIEN	triethylene tetramine
TGN	*trans*-Golgi network
TTM	tetrathiomolybdate
ULK1/2	autophagic proteins Unc-51-like kinase 1 and 2
